# Dexpramipexole alleviates chronic allodynia from sciatic neuropathy and reduces spinal interferon-γ and other proinflammatory cytokines in spinal cord and dorsal root ganglia in mice

**DOI:** 10.18103/mra.v14i5.7499

**Published:** 2026-05-31

**Authors:** Noah A. Castro, Clara J. Tomicek, Melody S. Sun, Justine R. Zimmerly, Annette K. Fernandez-Oropeza, Sammy D. Gomez-Medina, Zarena M. Dominguez, Shahani Noor, Jessie R. Maxwell, Erin D. Milligan

**Affiliations:** 1 Department of Neurosciences, University of New Mexico Health Sciences Center, Albuquerque, NM, USA; 2 Department of Pediatrics, University of New Mexico Health Sciences Center, Albuquerque, NM, USA

## Abstract

Dexpramipexole is a small molecule compound with properties previously shown to block the voltage-gated sodium channel, Nav_1.8_ on nociceptors. It additionally has anti-inflammatory properties in immune cells. In addition to clinical trials investigating the therapeutic efficacy of dexpramipexole to control eosinophilic asthma, dexpramipexole is effective in alleviating peripheral neuropathies in preclinical mouse models such as sciatic nerve chronic constriction injury, which reverses pathological sensitivity to light touch (i.e. allodynia). Pain signals are further processed in limbic areas of the brain that serve to regulate stress. Such overlapping regulation elicits increases in hypothalamic efferent sympathetic activity, which engages the spleen and adrenal glands in response to stress; organs critical in regulating immune responses and producing catecholaminergic norepinephrine, respectively. Thus, the current study evaluated the action of dexpramipexole (1) on the full timecourse of peripheral (intravenous) dexpramipexole and the acute timecourse of spinal (intrathecal) dexpramipexole on established allodynia from chronic constriction injury, and (2) characterized spinal and limbic proinflammatory cytokine protein expression following the spinal intrathecal dexpramipexole application in female mice. Splenic cytokine mRNA expression and adrenal norepinephrine levels were measured, as chronic constriction injury-induced neuropathy elicits elevated sympathetic nervous activity. Hindpaw sensitivity was assessed using the von Frey method. After intrathecal dexpramipexole and hindpaw reassessment at 1hr, the anterior cingulate cortex, amygdala, hypothalamus, dorsal lumbar spinal cord, lumbar dorsal root ganglia, spleen and adrenal glands were collected. Intravenous dexpramipexole, resulted in profound allodynic reversal within 1hr through 24hr, that returned by 48hr occurred. Intrathecal dexpramipexole, resulted in full reversal of allodynia by 1hr occurred. Chronic constriction injury-induced elevations in spinal interferon-γ protein was significantly suppressed from intrathecal dexpramipexole-treated mice that also demonstrated allodynia reversal, whereas trends for reduced spinal interleukin-1β and tumor necrosis factor-α were observed. Lumbar dorsal root ganglia from allodynic reversed mice given intrathecal dexpramipexole revealed reduced interleukin-1β and tumor necrosis factor-α, as was observed in the hypothalamus. Intrathecal dexpramipexole did not reliably alter splenic cytokine mRNA expression nor adrenal norepinephrine. These data suggest dexpramipexole may control allodynia by decreasing spinal interferon-γ and sensory ganglia interleukin-1β and tumor necrosis factor-α, which may serve as a potential pain therapeutic in addition to its action at Nav_1.8_.

## Introduction

Previous studies have shown that pro-inflammatory cytokines, such as interleukin-1beta (IL-1β) and tumor necrosis factor-alpha (TNFα), become elevated in the dorsal root ganglion (DRG) and spinal cord following induction of chronic neuropathies produced by a range of manipulations including the well-characterized constriction injury (CCI) of the sciatic nerve in mouse models ^1–3^. These pro-inflammatory cytokines are well-characterized to drive peripheral neuropathies such as thermal hyperalgesia and mechanical allodynia. For example, perineural endogenous overexpression of TNFα ^4,5^, or intramuscular application of TNFα results in thermal hyperalgesia and muscle allodynia, respectively ^4,5^. Additionally, spinal application (intrathecal, i.t.) of IL-1β or TNFα induces hindpaw thermal hyperalgesia and mechanical allodynia ^6^. The critical spinal role of TNF α in the maintenance of chronic neuropathy is supported by reports demonstrating that i.t. pharmacological inhibition of TNFα signaling using etanercept reverses allodynia in a mouse model of diabetic peripheral neuropathy ^7^. Moreover, i.t. administration of IL-1β can cause the development of allodynia and thermal hyperalgesia in the absence of a pathological injury ^8^, suggesting that spinal IL-1β signaling is required for the development of pathological pain following peripheral nerve damage in mice, as reported in a separate study ^9^.

Substantial evidence exists that the spinal actions of IL-1β and TNFα contribute to the development allodynia following peripheral nerve injuries, with other spinal cytokines having recently emerged that are also strongly implicated in pathological pain. Additionally, both mRNA and protein levels of the proinflammatory cytokine, interferongamma (IFN-γ) in the DRG and spinal cord have been shown to increase following CCI in both mice and rats ^10,11^. Previous work in rats with a monoarthritic model of inflammatory pain have shown that an i.t. injection of anti-IFN-γ antibody was able to attenuate mechanical allodynia, suggesting that spinal IFN-γ is necessary for the development of allodynia. In normal rats, allodynia could be induced by an i.t. injection of recombinant IFN-γ ^12^ demonstrating that spinal IFN-γ is sufficient in terms of inducing allodynia. IFN-γ expression levels are implicated in other mouse models of inflammatory pain, such as inflammatory bowel disease (IBD), where a gene knockout of IFN-γ receptors prevent the development of dextran sodium sulphate-induced colitis, body-weight loss, and increased mortality compared to controls ^13^. In rats following disc herniation, spinal application of IFN-γ increased nociceptive activity in the dorsal root ganglia ^14^. Following spared nerve injury (SNI), a model of sciatic nerve injury, adult rats develop robust allodynia along with elevated IFN-γ mRNA levels in the spinal cord. IFN-γ receptor-deficient mice did not develop the same hypersensitivity after SNI ^15^ linking IFN-γ as a necessary factor for driving chronic neuropathy from sciatic nerve damage.

Though the centrally-projecting terminals of nociceptors from DRG to the dorsal horn of the spinal cord are necessary for the evolution of neuropathic pain, nociceptive-responsive neurons (i.e. pain projection neurons) within the dorsal horn of the spinal cord posses ascending axons to various brainstem^16–19^ and limbic areas ^20^. Pain signals from the spinal cord can be substantially modulated at key limbic areas ^21^ that also serve other functions including stress regulation and response ^22^. Stress modulatory limbic areas that overlap with the processing of pain signals include the anterior cingulate cortex, amygdala and the hypothalamus ^22^. Thus, as part of the current study, a characterization of cytokines in limbic areas was conducted for changes in cytokine protein expression as a consequence of chronic CCI-induced allodynia and the potential impact that pain alleviation following therapeutic intervention may exert on such cytokine expression in these limbic areas.

The hypothalamus is a critical limbic area that integrates stress responses, as it functions to produce not only neuroendocrine output, but also the rapidly-acting autonomic response via projections to brainstem and spinal neurons that, via sympathetic fibers, act to maintain bodily homeostasis ^18,23,24^. Efferent sympathetic fibers of the autonomic nervous system are under the control of the hypothalamus and directly communicate to the spleen (via pre- and post-synaptic ganglia), which is a critical secondary lymphoid organ containing several types of immune cells and functions in regulating local and systemic innate and adaptive immune responses ^25^. Splenic immune cell morphology and cytokine expression are vulnerable to stressors, which may underlie disruptions in host immune responses following stress ^26^. Additionally, while the adrenal cortex is critical in the production of the stress-induced hormone, corticosterone, it is also influenced by the post-ganglionic innervation of efferent sympathetic fibers whose preganglionic neurons are directly innervated by the hypothalamus. Upon stress and sympathetic activation, the adrenal medulla rapidly produces the catecholamine, norepinephrine. Thus, adrenal norepinephrine can be an indication of real-time stress-induced sympathetic activity ^27^. In support, a prior report demonstrated that in rats, the sciatic nerve injury-induced stress response of the adrenal medulla included elevated norepinephrine ^28^. Therefore, the current study included a characterization of adrenal norepinephrine expression levels as an indicator of stress from chronic allodynia and whether altered norepinephrine (NE) could reflect changes in sympathetic activity after therapeutic intervention resulting in pain alleviation.

As pramipexole is a compound that selectively agonizes the dopamine (DA) D2/D3 receptors ^29,30^ and is FDA-approved for long-term treatment of Parkinson’s Disease ^31^, Dexpramipexole (DPX) is the R(+) enantiomer of pramipexole, and unlike pramipexole, is virtually devoid of DA receptor agonist actions ^32,33^. DPX has high oral bioavailability, is water-soluble, and rapidly achieves and maintains high central nervous system (CNS) concentrations relative to plasma ^34^. In a preclinical report, intravenous (i.v.) DPX reversed allodynia in female mice induced by CCI, and in the same report, demonstrated that stimulated peripheral immune cell treatment with DPX dose-dependently increased gene expression of the anti-inflammatory cytokine, IL-10 ^35^. However, DPX is shown to bind the specific voltage-gated sodium channel Nav_1.8_^36^ that is present on peripheral and spinal central terminals and cell bodies of primary nociceptors in mice and humans ^37^. Peripheral administration of DPX in experimental mice demonstrates activity as a neuronal Nav_1.8_ antagonist and acutely reduces the nociceptive behavior induced by inflammatory pain from hindpaw formalin injection or induction of ankle arthritis, and in neuropathic pain models such as mouse sciatic nerve damage, chemotherapy- and diabetic-induced neuropathy ^36^. The promise of targeting Nav_1.8_ with a specific antagonist for pain control was demonstrated in a phase 2 clinical trial that reported oral adminsitration of the highly-selective Nav_1.8_ antagonist, VX-548, resulted in *modest* pain reduction in 60% of particiants, while only 25% of participants reported ~ 70% reduction following acute surgery (abdominoplasty or bunionectomy) during a 48-hour treatment period ^38^. Thus, the clinical therapeutic effects of selectively targeting Nav_1.8_ further support that controlling pain requires engaging mechanisms beyond only targeting Nav_1.8_, and increased therapeutic benefit could be accomplished by also impacting the aforementioned proinflammatory factors.

Therefore, the strong preclinical link of activated glia and proinflammatory cytokine actions to the development and maintenance of pathological pain, and the possible additional mechanism of DPX exerting anti-inflammatory processes, the goals of the current study that aimed to examine DPX efficacy *in vivo* for pain control were four-fold, to: (1) validate prior work demonstrating the magnitude and duration of reversal of nerve injury-induced chronic allodynia following i.v. DPX in female mice, (2) determine whether the *in vivo* action of DPX on allodynia reversal could occur by blunting the pronociceptive cytokines, IL-1β, TNFα and IFN-γ at the level of the lumbar dorsal spinal cord or lumbar DRG when administered locally via the i.t. route, (3) characterize the cytokine expression profile in limbic areas that regulate stress (anterior cingulate cortex, amygdala and hypothalamus), and (4) characterize allodynia-mediated sympathetic responses of splenic cytokine gene expression (interleukin-10, IL-1β and TNFα) and adrenal norepinephrine levels. Examining DPX’s effects on allodynia via different routes of administration, and consequent cytokine expression in the spinal cord, DRG, and limbic regions may provide further insight into DPX’s breadth of action and may expand the clinical scope of pain therapeutics available for treating neuropathic pain states.

## EXPERIMENTAL PROCEDURES

### Animals

All procedures adhered to the ethical guidelines for laboratory animals in research (Council, N.R, 2011), which required approval by the Institutional Animal Care and Use Committee (IACUC) of the University of New Mexico Health Sciences Center, and are reported according to ARRIVE Guidelines (Kilkenny et al., 2010). All mice were housed in groups of 4 mice/cage in a temperature (23° ± 2 °C) and light-controlled (normal 12hr:12hr light/dark; lights off at 19:00h) rooms, with standard rodent chow and water available *ad libitum* prior to and during experimentation. All procedures closely adhered to guidelines from the International Association for the Study of Pain for the use of animals in research (Foundation for Biomedical Research, The Biomedical Investigator’s Handbook for Researchers Using Animal Models. Washington, D.C.: FBR, 1987. WWW: https://www.fbresearch.org/).

The current report utilized a total of 47 female mice at 7–11 wks, with N=24 allotted for i.v. DPX treatment and N=23 for intrathecal (i.t.; peri-spinal) DPX treatment. Mice were purchased from Jackson Laboratories (wildtype; FFID: IMSR_JAX:000664, Bar Harbor, ME, USA) at ~ 16–23 g to examine the effects of DPX on allodynia when given by different routes of administration; either by i.v. or i.t.delivery.

### Chronic Constriction Injury of the Sciatic Nerve

This study followed the chronic constriction injury (CCI) model procedure as previously detailed ^35^. Briefly, mice were placed under isoflurane anesthesia (1.5–2.0% volume in oxygen, 2.0 L/min) and the left thigh was shaved and cleaned with 70% ethanol. Once the area was dry, a small incision with a #15 blade was made over the posterior thigh after palpating the femur, (~5 mm the femur), and the muscle fascia of the mouse was exposed. Using sterilized blunt dissection scissors, the overlying muscle gently separated, and sterile plastic probes were used to gently isolate the sciatic nerve, and irrigated with sterile isotonic saline to prevent dehydration of the nerve. Animals receiving the CCI underwent ligation of three, 5–0 chromic gut sutures around the left sciatic nerve, assuring that the sutures were not pinching the nerve and sufficiently loose to gently slide the suture along the nerve. Animals receiving the sham control surgery underwent the same procedure but did not receive chromic gut ligation. Following manipulation, the nerve was tucked back into its initial area, and overlying muscle was closed using two 4–0 sterile silk sutures. Two wound clips were then used to close the overlying skin. Mice were closely monitored to determine full recovery from surgery and anesthesia (~5 min). Throughout the entire study, 1 mouse demonstrated complications from the surgery and was promptly excluded from the study by euthanasia, resulting in N=23 for the i.t. DPX experiment. All other mice recovered completely from the surgery and anesthesia without any complications.

### Behavioral Assessment of Allodynia

Behavioral assessment of allodynia was done exactly as highlighted previously with minor modifications ^35^. Briefly, following a 7 -day acclimation to the animal colony room, the female mice were subjected to a 1 hr habituation period where they were placed atop a graded testing rack placed along the wall within the colony room at the opposite wall where their home cages were positioned. The rack consisted of bars spaced 1 cm apart, which allowed full access to the plantar surface of the hindpaws to induce paw withdrawal responses ^35^. The habituation period was approximately 1 hr/day for 4 sequential days conducted within the first 4 hours of the light cycle (Lights on from 0700 hrs to 1900 hrs) in a sound and temperature (23° ± 2 ° C) controlled environment.

Hindpaw responses to light touch stimuli was tested by using the von Frey behavioral assay in which a series of calibrated monofilaments were applied to the hindpaws to illicit a paw withdraw response. The series of calibrated monofilaments were applied to the mouse right and left hind paw for a maximum of 3 seconds, allowing 30 seconds between testing the same mouse again. The log intensity of the eight monofilaments used is defined as log10 (grams ×10,000) with the range of stimulus intensity as follows: (reported in log (grams)), 2.36 (0.022 g), 2.44 (0.028 g), 2.83 (0.068 g), 3.22 (0.166 g), 3.61 (0.407 g), 3.84 (0.692 g), 4.08 (1.202 g), and 4.17 (1.479 g). Testing began with the fiber 3.22, which was in the middle of the assay scale. All testing procedures were conducted as previously described ^35^. Following behavioral analysis, the total number of responses/non-responses to the von Frey fibers was entered into the computer program software, PsychoFit (https://psych.colorado.edu/~lharvey: RRID: SCR_015381) to determine the absolute withdrawal threshold (50% paw withdrawal threshold), as previously described ^35^. Data were plotted in GraphPad Prism (Prism 10; Version 10.6.1). Hindpaw threshold assessment was performed prior, at baseline (BL), and days 3, 7, 10, 14 post-surgery. After Day 14 post-surgery, hindpaw thresholds were assessed according to that detailed in the graphs reflecting behavior after i.v. DPX and i.t. DPX.

### Drug Preparation

Drug preparation was done as previously reported with minor modifications (35). Briefly, DPX was reconstituted with 0.9% isotonic saline and aliquoted into 1:1 aliquots of a 1 μg/μL stock solution. The drug was then stored in sterile Eppendorf tubes sealed with parafilm and frozen at −80° C for future use. On injection day, aliquots were allowed to fully thaw at room temperature and subsequently diluted to a 0.1 μg/μL concentration using 0.9% isotonic saline, and vortexed for approximately 15 s. The volume of each i.v. injection was held constant at 50 μL. The volume of each i.t. injection was held constant at 5 μL. The vehicle of either the i.v. (50 μL) or i.t. (5 μL) mice was 0.9% sterile isotonic saline. Animals were injected within an hour of drug and injection preparation. Half of the mice were randomly assigned to either the drug group or the vehicle group.

### Intravenous Injection

DPX or veh was injected into the mouse tail vein using the same procedure as described previously ^35^, with injections performed on day 14 post-surgery within 2.5 hrs of lights ON. The experimental design was a 2 (sham vs. CCI) × 2 (DPX vs. vehicle) with N=6 in each experimental group. Intravenous administration was chosen to validate results from our prior report ^35^. Using aseptic techniques, 50 μL of DPX or veh was drawn up into individual 1 cc, 27G-5/8 insulin syringes and injected in to the lateral tail vein of mice that were placed in a clean, plexiglass platform specifically created for tail-vein injection and/or blood collection without restraint (Braintree Scientific, Inc., Cat# IL-300). The tail held firmly allowed for needle-tip insertion into the lateral tail vein ensuring blood efflux into the hub of the syringe occurred, followed by drug injection for 10 seconds. Following the injection, sterile gauze were placed on the tail to mitigate excess bleeding and subsequently, mice were placed back into their original home cage and monitored for the next 10 minutes for any adverse reactions. No adverse effects were observed in these mice. The average time for the handling and injection was between 2–3 minutes per mouse. The experimental conditions of these mice are represented in the table, ‘Experimental Groups’.

### Intrathecal Injection

Intrathecal (i.t.) injections were administered under isoflurane anesthesia after behavioral assessment of allodynia on day 14 post-surgery and was conducted similar to that previously described with slight modifications (39). Briefly, previously prepared injector units were assembled with two sterile 27G × 0.5 needles (PrecisionGlide, Becton Dickinson & Co.; Cat#:305109), one inserted into a 30 cm long unit of PE20 polyethylene tubing (Becton Dickinson & Co; Cat#:427406), with the opposite end containing another inserted 27G × 0.5 needle, but with the plastic needle hub removed such that the blunt end of the needle was then inserted into the PE20 tubing and the exposed beveled end of the needle remained available for the i.t. injection. These injector units were stored in a sterile, dry container until needed for i.t. injections. One injector per mouse was created. At the time of injection, a 50 μL calibrated glass Hamilton syringe was filled with sterile 0.9 % isotonic saline that was connected to the intact hub of the injector unit followed by plunging the sterile 0.9 % isotonic saline through the entire length of the PE20 injector unit and out of the exposed beveled needle end, thus creating a completely filled PE20 tubing of the injector unit. The 50 μL Hamilton syringe was then replaced with a 10 μ L Hamilton syringe creating an air bubble-free injector unit that was filled with saline. A 1 μL air bubble was then created in the injector unit at the exposed beveled needle end, and with the beveled needle end, then drawing up either 5 μL of the vehicle (0.9 % sterile isotonic saline), or DPX. Under 2% isoflurane anesthesia (oxygen at 2.0 L/min) mice were briefly shaved at the mid- to lower-lumbar region, lightly swabbed with 70% ethanol, and using the exposed beveled needle tip, inserted the needle tip between lumbar vertebrae 5 and 6 (L5-L6). A tail flick in the mouse was considered indicative of a successful i.t. placement, which was followed by drug or veh injection by plunging the contents of the injector unit using the attached 10 μL Hamilton syringe. This administration of the drug or vehicle injection was done over the course of 30 seconds. Immediately after injection, mice were allowed to recover from anesthesia and monitored for adverse effects for 10 minutes. All mice responded normally to the injections and no adverse reactions were reported. Total handling time of each mouse was about 3 minutes, which included anesthesia induction and injection. The experimental conditions of these mice are represented in the table, ‘Experimental Groups’.

**Table:** 

Experimental Groups	
i.v. DPX	
SHAM Veh n=6	SHAM DPX n=6
CCI Veh n=6	CCI DPX n=6
i.t. DPX	
SHAM Veh n=6	SHAM DPX n=6
CCI Veh n=5	CCI DPX n=6

### Tissue Collection

Tissue collection was conducted in four cohorts of mice (n=6 mice for 3 cohorts and n=5 for the remaining cohort). One hour after the i.t. injection and immediately following behavioral testing, mice were deeply anesthetized using isoflurane (10 min, 5% volume in oxygen at 2 liters per minute) followed by transcardial perfusion with ice-cold 0.1 M phosphate buffered saline (pH=7.40), flow rate was 5 mL/min for the initial 30 seconds, followed by10 mL/min for the remaining 3 minutes, as described previously ^39^. Blood clearance from the lungs and liver were indications of effective perfusion. Dissection of the lumbar (L3-L6) dorsal spinal cord ipsilateral to sciatic CCI and ipsilateral dorsal root ganglia (DRG), was similar to that previously described in Sleigh, Weir and Schiavo ^40^, as well as the spleen and adrenal glands Utilizing the Paxinos and Franklin atlas for mouse brain as a training guide, previously trained experimenters rapidly performed brain removal and dissection of the hypothalamus (HYP), amygdala (AMG), hippocampus (HIPP), anterior cingulate cortex (ACC) and frontal cortex (FC) on wet ice. All tissue samples were immediately placed into DNase/RNase/Protease-free 1.5 mL tubes (VWR; Cat #20170–026) on dry ice until stored at −80°C for future analysis.

### RNA Extraction and PCR Analysis of mRNA

Total RNA was extracted for spleen and DRG samples as previously described (42) using the miRNeasy Mini Kit (Qiagen; Cat. #217004) and miRNeasy Micro Kit (Qiagen; Cat. #217084), respectively, per manufacturer’s instructions. Briefly, frozen tissues were retrieved on dry ice before transferring into homogenization tubes. Spleen samples were transferred to a petri dish on dry ice for cutting with a metal spatula to ensure tissues remained frozen before homogenization. Petri dish, cutting blade, and metal spatula were cleaned with 70% ethanol between samples. Homogenizations were performed using a motorized VWR Disposable Pellet Mixer and cordless motor pestle system (VWR International; Cat#:47747–3). The entire DRG sample was processed as collected. Qiazol (200 μL) was added to homogenization tubes containing the frozen tissue, homogenized on ice for 90s followed by further addition of Qiazol (500 μL), vortexed for 30s, and incubated at RT for 7 min, before adding 140μL of chloroform (Sigma-Aldrich; Cat#C2432). Samples were shaken to mix for 15 sec, incubated for 4min at room temperature (RT), shaken vigorously for 10s, and then centrifuged at 4°C at 12000 × *g* for 15 min. Approximately 350 μl of the aqueous layer was retrieved from the samples and the final elution of RNA was in 40 μl (spleen) or 20 μL (DRG) of RNAse/DNase free sterile water. RNA concentration was assessed using the NanoDrop (Thermo Scientific, MA, United States).

Extracted RNA was diluted to a standardized concentration of 122ng/μL (±4ng/μL) for spleen samples and 40ng/μL (±4ng/μL) for DRG samples. Total RNA reverse transcribed for cDNA was 1440 ng for the spleen and 400 ng for the DRG. A lower amount of total RNA was loaded for the DRG samples due to the nature of the small tissue size. cDNA synthesis was performed using SuperScript^™^ IV VILO^™^ cDNA Synthesis Kit (Invitrogen; Cat#:11754250) per manufacturer’s instructions. cDNA was amplified with Taqman Gene Expression Assays (cat# 4351370, ThermoFisher Scientific), (Table 1).

The housekeeping gene used as a sample standard was 18s and was diluted to a factor of 1:800 in spleen samples and 1:40 in DRG samples due to its high abundance in cells. In the spleen samples, a 1:3 dilution was used for IL-1β, TNFα, and IL-10. The DRG samples were undiluted for IL-1β, TNFα, and GFAP due to their lower total RNA concentration yield. All targets were run in triplicate, and data are presented as 2ĈTnorm/2ĈTtarget as previously detailed ^41,42^.

### Protein Extraction

Protein was extracted from the DSC, AMG, HYP, and ACC by addition of a lysis buffer as previously detailed ^43^, containing phenylmethanesulfonyl fluoride solution (PMSF; Sigma Cat# 93482), Sodium Orthovanadate, Okadaic Acid, and freshly added protease and phosphatase inhibitors (Roche PhosStop Tablet; Cat # 04906837001, Mannheim, Germany), resulting in an optimal solution for extraction. Samples were homogenized for 1 min using a motorized homogenization system (VWR International; Cat#:47747–3), followed by 10 min of sonication. Tissue samples were centrifuged at 14,000 RPM at 4°C to pellet cellular debris, and the supernatant was collected and stored at −80°C. Protein concentrations were determined by QuickstartTM Bradford Protein Assay (BioRad; Cat#:500–0201). Protein extraction for the HIP, frontal cortex (FC, and adrenal glands was prepared as above with the exception that there was no homogenization, and the sonication was done for three cycles of ON:OFF alternating 30 sec utilizing the Biorupter Plus sonication device (Diagenode; Cat #B01020014) according to protocol. This change was implemented to prevent protein degradation in delicate tissue regions, which was not previously accounted for in protocols. Protein expression levels were then determined using a V-plex multiplex electrochemiluminescent immunoassay platform on AMG, HYP, DSC, and ACC brain tissues on the following 10 cytokines: IFN-γ, IL-1β, IL-2, IL-4, IL-5, IL-6, IL-10, IL-12p70, KC/GRO (a.k.a. CXCL1), and TNFα (Meso Scale Diagnostics; Cat. #K15225D). Assays were performed as previously described, following a similar approach to human studies ^43–45^. Tissue lysate was added undiluted to the multi-spot plate up to a volume of 50 μL and incubated at 4°C overnight while shaking. Assay was performed according to the manufacturer’s instructions and read on a Quickplex SQ 120 Imager (MesoScale Discovery, Gaithersburg, MD, United States). Most samples were run in singlet. Coefficient of variation for duplicate samples was less than 13% for AMG and ACC, 33% for HYP, and 42% for DSC across all targets.

### Enzyme-linked Immunosorbent Assays

Assay was performed on the adrenal glands using the Abcam Norepinephrine (NE) enzyme-linked immunosorbent assay (Abcam; ELISA Kit, Cat. # ab287789). Assay was performed according to the manufacturer’s instructions. Briefly, a six-point standard curve was formed using the lyophilized standard in the range of 0–1000 pg/mL. Samples were added undiluted in equivolume to wells, along with the Biotin-detection antibody, and incubated for 45 min. After washing wells three times with wash buffer, HRP-Streptavidin Conjugate (SABC) was added, and the plate was incubated for 30 min. The plate was washed 5 times with wash buffer, and TMB substrate was added prior to another 30-min incubation. The stop solution was added, and the plate was read at 450 nm immediately thereafter. Duplicate standards and singlet samples (50uL/well) were prepared. Relative NE was acquired according to the standard curve generated and the amount of total protein loaded into the wells.

### Statistical Analysis

All statistical analyses were performed on GraphPad Prism version 10.2.3 (GraphPad Software Inc., San Diego, CA, United States). Ipsilateral hindpaw sensitivity thresholds were conducted separately from contralateral hindpaw sensitivity thresholds. A one-way analysis of variance (ANOVA) was applied for differences between surgeries (CCI vs. Sham) at baseline (BL). A two-way repeated measures ANOVA was applied to identify hindpaw threshold differences after CCI of Sham, but before either i.v. or i.t. injection (i.e. CCI vs. Sham) from BL to Day 10. A three-way ANOVA was performed to assess differences between surgery (CCI vs. Sham), injection type (Veh vs. DPX), and time from Day 14 to after injection.

Analyses of protein and mRNA cytokine expression were conducted by applying a two-way ANOVA, followed by applying the original false discovery rate (FDR) method of Benjamini and Hochberg ^46^ for multiple group comparisons. The threshold for statistical significance was set *a priori* at *p p*≤0.05 for all comparisons. Outliers were removed following Grubbs’ *Z*-test ^47^. All graphics data are represented as mean ± standard error of the mean (SEM).

## RESULTS

### Intravenous dexpramipexole fully reverses chronic construction injury-induced allodynia in mice

**Figure 1:**
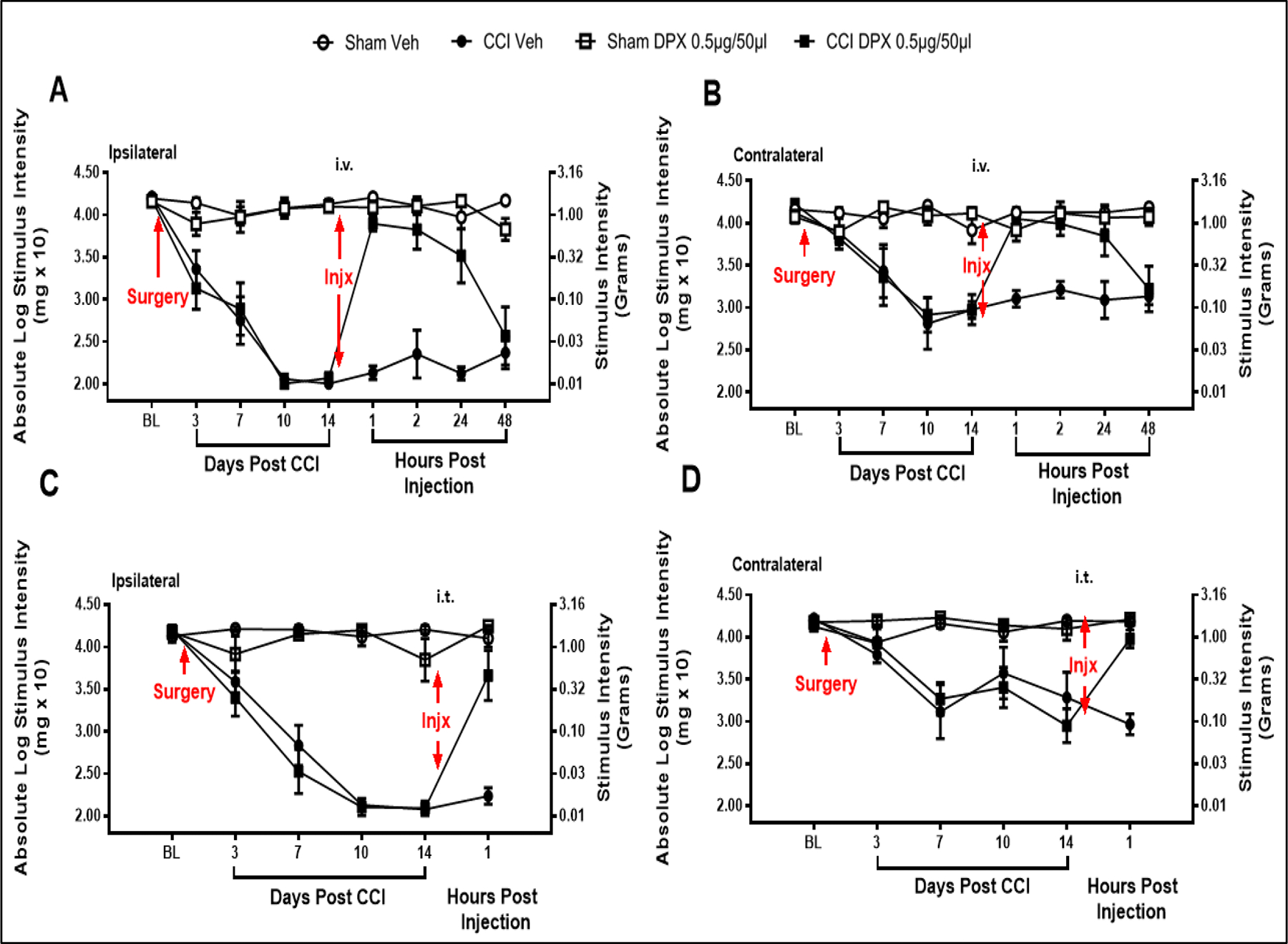
Dexpramipexole given by systemic (intravenous; i.v., 0.5μg/50 ul via tail vein) or spinal (intravenous; i.v., 0.5 μg/10 ul) administration, relative to vehicle (veh), fully reverses chronic constriction injury (CCI)-induced hindpaw allodynia in female mice. Behavioral responses shown for light touch stimuli (grams) applied to the plantar surface of the ipsilateral (**A, C**) and contralateral (**B, D**) hindpaw. The stimulus threshold for all groups is similar at baseline (BL).

The stimulus threshold for all groups represented in Fig A-D is similar at baseline (BL) prior to the sciatic nerve injury. For the experiment examining i.v. dexpramipexole on chronic allodynia, hindpaw responses at BL displayed similar sensitivity (ipsilateral: F_1,20_ = 0.5, p=0.4871; contralateral: F_1,20_ = 0.8, p=0.3685) (Fig. 1A & B). Following surgical manipulation, sham groups show stable responses that were similar to BL thresholds through Day 14, whereas mice with CCI manipulation developed bilateral hindpaw sensitivity reaching maximal levels by Day 14 (Fig 1A & 1B), with a main effect of time (ipsilateral: F_3.807, 76.13_ = 21.67, p< 0.0001; contralateral: F_3.734, 74.68_ = 7.779, p < 0.0001), surgery (ipsilateral: F_3, 20_ = 121.7, p< 0.0001; contralateral: F_3,20_ = 40.34, p < 0.0001), and an interaction between time and surgery (ipsilateral: F_24,160_ = 10.98, p < 0.0001; contralateral: F_24,160_ = 4.238, p < 0.0001). This pattern of bilateral sensitivity replicates prior reports ^48^. Immediately following behavioral testing on Day 14, mice received injections of 0.5 μg dexpramipexole (DPX) by either intravenous (i.v.) tail vein or equivolume saline (50 ul), or (C, D) intrathecal (i.t) route or equivolume saline (10 ul) as vehicle. After DPX administration on Day 14, compared to mice that received i.v. vehicle (saline), mice that received i.v. dexpramipexole (DPX) demonstrated reversal from allodynia as assessed at 1, 2 and 24 hours post injection, with allodynia returning by 48 hours, as revealed by a main effect of DPX treatment (ipsilateral: F_1,50_ = 50.86, p<0.0001; contralateral: F_1,50_ = 18.40, p<0.0001), time (ipsilateral: F_4,50_ = 9.967, p< 0.0001; contralateral: F_4,50_ = 5.588, p< 0.0001) and of CCI surgery (ipsilateral: F_1,50_ = 349.9, p<0.0001; contralateral: F_1,50_ = 109.2, p<0.0001), with interactions between CCI surgery and DPX treatment (ipsilateral: F_1,50_ = 48.06, p < 0.0001; contralateral: F_1,50_ = 16.21, p< 0.0002).

### Intrathecal dexpramipexole fully reverses chronic construction injury-induced allodynia in mice

At BL, all groups of mice displayed similar hindpaw sensitivity thresholds (ipsilateral: F_1,19_ = 0.9, p=0.3437; contralateral: F_1,19_ = 0.1, p= 0.6814) (Fig 1C & D), replicating findings shown in Figure 1A & B). Compared to Sham-treated mice that remained stable and near BL values, mice that underwent CCI displayed heightened bilateral hindpaw sensitivity that developed during a 7-day period and remained relatively stable through Day 14 post-sciatic nerve injury, as supported by a main effect of time (ipsilateral: F_3.778,71.77_ = 33.85, p<0.0001; contralateral: F_2.939_, _55.84_ = 8.588, p<0.0001), surgery (ipsilateral: F_3,19_ = 165.2, p<0.0001; contralateral: F_3,19_ = 27.15, p<0.0001), and an interaction between time and surgery (ipsilateral: F_15,95_ = 14.30, p<0.0001; contralateral: F_15,95_ = 5.245, p<0.0001). Following hindpaw assessment on Day 14, mice treated with CCI and that received i.t. vehicle (saline) demonstrated persistent allodynia throughout the remainder of timecourse, while i.t. dexpramipexole (DPX) induced a profound reversal of allodynia that was measured at 1-hour post injection, as revealed by a main effect of CCI surgery (ipsilateral: F_1,54_ .= 533.3, p<0.0001; contralateral: F_1,54_ = 125.0, p<0.0001), time (ipsilateral: F_5,60_ = 29.54, p<0.0001; contralateral: F_5,60_ = 7.084, p<0.0001), and an interaction between surgery and drug ipsilaterally (ipsilateral: F_1,54_ = 4.755, p=0.0336; contralateral: F_1,54_ = 0.3759, p=0.5424).

### Intrathecal dexpramipexole treatment significantly suppresses spinal interferon-γ protein levels

**Figure 2:**
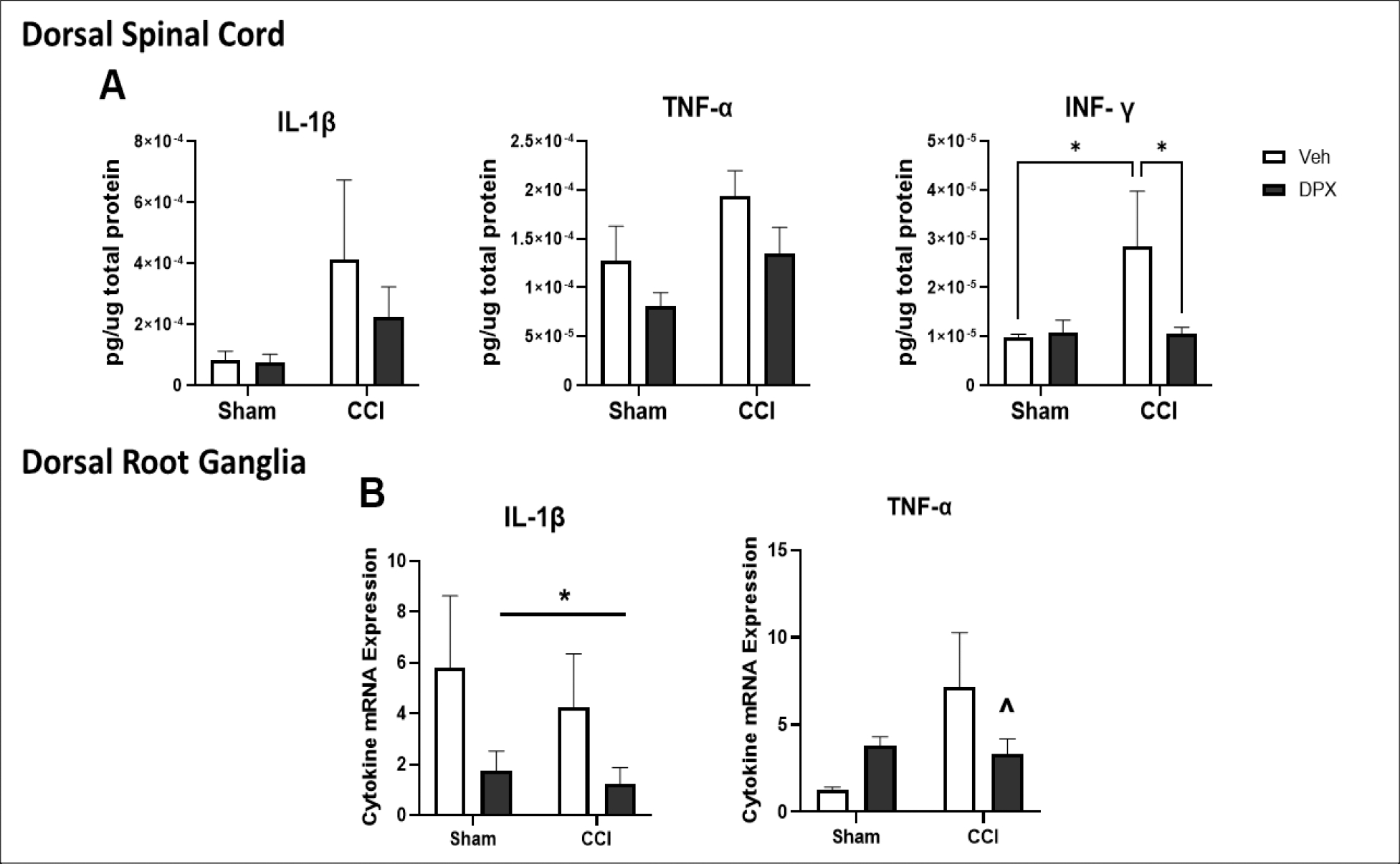
Intrathecal dexpramipexole (DPX) in mice with chronic constriction injury (CCI) result in decreased pronociceptive cytokine levels; interleukin-1beta (IL-1β), tumor necrosis factor-α (TNF-α) and interferon gamma (IFN-γ). **(A)** Protein expression levels in picogram (pg) target protein/microgram (ug) total protein in the dorsal spinal cord. **(B)** Expression of mRNA levels. * DPX main effect, ^ DPX and CCI interaction denote p≤0.05.

While sham-treated mice that received either an i.t. saline or DPX injection revealed similar protein levels of interferon-γ (IFN-γ), mice with CCI given i.t. saline revealed significantly elevated levels of IFN-γ protein in the dorsal spinal cord compared to mice with CCI who received i.t. DPX, as revealed by an interaction of CCI and DPX (F_1,12_ = 4.540, p = 0.054). (p=0.0207), and (Fig 2A). Levels of pro-nociceptive cytokine IL-1β show modest but insignificantly increased levels in CCI mice who received the saline injection compared to their Sham counterparts (p=0.1492), (Fig 2A). However, levels of pro-nociceptive TNFα are significantly increased in the dorsal spinal cord following CCI treatment, as revealed by a main effect of sciatic manipulation (F_1,17_ = 4.599, p=0.0467). Although not significant, a trend toward decreased expression of TNFα following administration of i.t. DPX in both CCI- vs. sham-treated (p = 0.1646; p = 0.2523, respectively) (Fig 2A). There were no reliable differences in protein cytokine expression of other cytokines analyzed for the dorsal spinal cord.

### Dexpramipexole blunts cytokine mRNA expression in the dorsal root ganglia

Unexpectedly, IL-1β mRNA expression was elevated in mice that received sham manipulation with i.t. saline, which is an important control group that completely lacked hindpaw sensitivity (Fig 2B). The gene expression levels were similar to that observed in mice with CCI given i.t. saline. These data suggest that the i.t. injection itself caused local inflammation that was not sufficient to impact nociceptor excitability and communication. However, in support of an anti-inflammatory function, DPX blunted mRNA expression of IL-1β in mice with either sham or CCI manipulations, which was supported by a main effect of DPX (F_1,17_ = 4.456, p= 0.0499) (Fig 2B). In consideration of the allodynic profile of CCI-treated mice given i.t. vehicle, the pro-inflammatory cytokine, TNFα, may be playing an additional role, as prior published work demonstrates. The current data show that while an increase in TNFα is demonstrated in mice following CCI (F_1,15_ = 3.2, p = 0.09), mRNA expression of TNFα is significantly blunted in mice with CCI and treated with DPX, as supported by an interaction of sciatic neuropathy and drug treatment (F_1,15_ = 4.553, p = 0.0498) (Fig 2B). It is notable that sham-treated mice given i.t. saline revealed the least amount of mRNA expression for TNFα compared to CCI mice treated with i.t. saline-treated (p = 0.02).

### Intrathecal dexpramipexole blunts interleukin-1β and tumor necrosis factor-a protein expression in the hypothalamus

**Figure 3:**
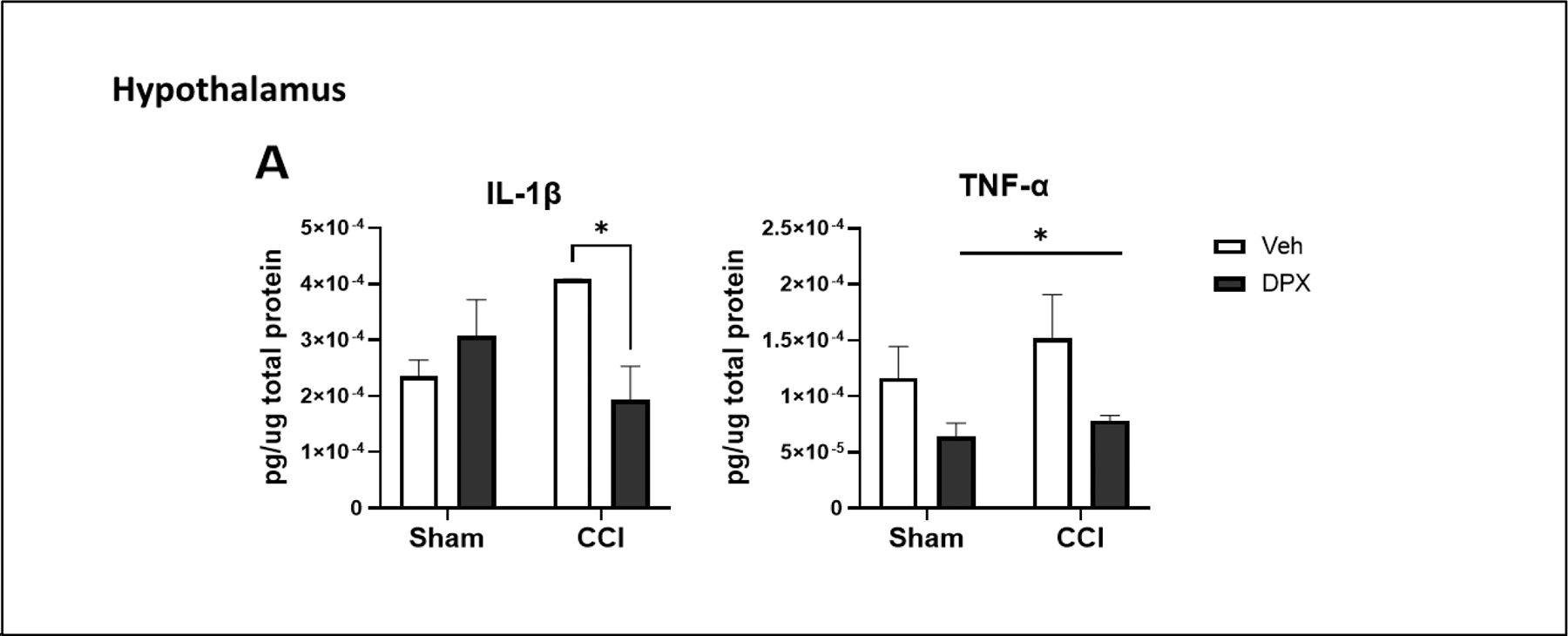
Protein expression levels in picogram (pg) target protein/microgram (ug) total protein in the hypothalamus. **(A)** Levels of the pro-nociceptive cytokine, interleukin-1**β** (IL-1 **β**), and are decreased in chronic constriction injury (CCI)-treated mice given dexpramipexole (DPX) vs. vehicle (veh), or **(B)** reduced tumor necrosis factor-**α** (TNF-**α**) given DPX regardless of CCI. N=3–5/group. For panels A and B, * = p ≤0.05.

The hypothalamus is a structure that is linked to modulating pain signals at the level of the spinal cord. We show that mice treated with i.t. DPX and CCI demonstrated significantly reduced IL-1β protein levels, as supported by an interaction between sciatic nerve manipulation and DPX treatment (F_1,12_ = 5.552, p=0.0363) (Fig 3A). Further, CCI-treated mice given i.t. veh revealed greater IL-1β protein levels relative to sham-treated mice with i.t. veh and CCI-treated mice with i.t. DPX (p=0.0953, p=0.0232, respectively) (Fig 3A). In addition, significantly lower levels of TNFa were measured in mice with either sham or CCI (F_1,10_ = 7.748, p= 0.0193) (Fig 3A), with significantly blunted levels measured in CCI-treated mice with i.t. DPX when compared to their saline control counterparts (p=0540). In the anterior cingulate cortex, IL-6 protein levels were reduced in CCI + DPX treated mice. Data of other modest changes in cytokines levels from either CCI and/or DPX treated are presented in Table 2

### Dexpramipexole and sciatic chronic constriction injury do not alter mRNA or protein expression in peripheral tissues

No reliable changes were detected between treatment groups in the spleen mRNA expression levels for IL-1β, TNFα, or IL-10. No reliable changes due to DPX or CCI were detected in protein norepinephrine levels in the adrenal glands (Table 3).

## DISCUSSION

It is well-documented in a variety of animal models of peripheral neuropathies that pro-nociceptive cytokines such IL-1β and TNF-α exert critical roles in generating increased activation of nociceptor cell bodies in the DRG along with their increased communication to pain transmission neurons in the dorsal spinal cord, thus supporting the goal of identifying therapeutics capable of controlling such cytokines. For example, a study utilizing conditional knockout mice that lacked the gene for TNF receptors showed no development of thermal hyperalgesia and lessened mechanical allodynia following CCI when compared to wild-type controls ^49^. Further, a conditional knockout of IL-1 receptors in mice showed a delayed onset of and faster recovery of tactile allodynia compared to controls ^50^. In other studies, epineural application of neutralizing antibodies to TNF**α** prevented the development of allodynia in a CCI mouse model when administered before the induction of nerve injury ^51,52^. The findings from the current report showing a reduction of IL-1β mRNA levels in the DRG following DPX application is congruent with these prior works.

Attempts to utilize compounds that exert anti-inflammatory actions support the notion of targeting cytokines for pain control. For example, one study showed that intervention with Metamizole, which is a small molecule compound with pleiotropic effects that also alleviates pain and has some anti-inflammatory properties, reversed allodynia and reduced DRG cytokine expression in rats with CCI ^53^. Other compounds that impact the inflammatory milieu include DPX, which has been examined as a potential therapeutic given its high safety profile. Utilizing the well-characterized animal model of peripheral neuropathy, the current report confirmed elevated TNF**α** mRNA levels in the DRG following CCI, thus confirming such outcomes demonstrated in previously documented work in both rats and mice ^54,55^. Notably, data reported in the present report show DPX treatment reduced TNF-α expression in the DRG of mice with CCI, albeit a modest and non-significant trend. In the spinal cord, a trend of TNFα and elevated IL-1β protein levels were reduced following DPX application.

Several other compounds have been examined in the preclinical setting to control allodynia/hyperalgesia that are linked to suppressing IL-1β and TNFαThe drug, sulforaphane (SFN), which is a small molecule phytochemical containing anti-oxidant properties and studied in a CCI mouse model effectively reduced IL-1β and TNFα mRNA and protein levels from the injured sciatic nerve. Notably, the presence of peri-sciatic proinflammatory cytokines is thought to be due to immune cells that extravasate to the sciatic nerve. Sulforaphane was administered with repeated intraperitoneal (7 days) injections immediately following induction of CCI. The reduction in IL-1**β** and TNF**α** was accompanied by a reduction in mechanical and thermal allodynia ^56^. A separate study in rats with bone cancer-induced allodynia demonstrated similar reversal of hyperalgesia and reduction of spinal proinflammatory cytokines following i.t. injection of SFN ^57^. These studies support that controlling spinal proinflammatory cytokine actions may also control allodynia induced by peripheral nerve pathology. Resveratrol is another therapeutic that is present in foods such has berries and nuts with anti-oxidant and anti-inflammatory properties ^58^, and in preclinical studies, has been shown to reduce IL-1β as well as TNF**α** mRNA and protein levels in the sciatic nerve in a dose dependent manner in CCI mice ^59^. The reduction of allodynia was best achieved when resveratrol was administered interperitoneally seven days after surgery, at peak sensitivity. Together, these data support the notion that the development of tactile hypersensitivity such as allodynia which is accompanied by increased levels of proinflammatory cytokines, can be targeted by drug therapeutics that act to reduce proinflammatory cytokine levels. It is important to consider the clinical aspect of these drugs via systemic application as both drugs were able to cross the blood-brain barrier, offering less invasive delivery mechanisms.

Dexpramipexole in clinical practice is a promising therapeutic used for hypereosinophilic syndromes. It is effective in reducing eosinophilic levels in asthma patients, while having minimal adverse effects ^60–62^. A clinical trial of adults who had inadequately controlled moderate to severe asthma with elevated eosinophil counts in their blood found that effective eosinophil lowering occurred following DPX treatment ^61^. Eosinophils are innate immune cells that play a critical role in clearing parasitic infections, but are capable of generating tissue damage accompanying allergic responses through proinflammatory processes, including asthma ^63^, which DPX controls by acting to inhibit eosinophilic development in the bone marrow ^61^. Additionally, DPX has been shown to create neuroprotective effects by reducing reactive oxygen species and increasing mitochondrial ATP production in neuronal and glial cell cultures ^64^. Unlike its enantiomer, pramipexole, DPX has minimal dopaminergic activity ^32^, and offers similar reversal of allodynia in a neuropathic pain model, thus supporting the rationale to further investigate DPX as a pain therapeutic by controlling potential anti-inflammatory actions. The data from the present report show that following CCI, mice exhibited bilateral allodynia beginning on day 3 post-surgery and reached maximum tactile sensitivity by day 10 post-surgery, with DPX administration robustly reversing allodynia. This effect was seen when administered systemically or intrathecally, indicating that more than one underlying mechanism is responsible for the anti-allodynic effects of DPX. Specifically, it is likely that DPX is acting at Nav_1.8_ channels on nociceptors, as previous work has demonstrated in several mouse models of chronic neuropathy ^36^. However, in addition to its peripheral actions, DPX may likely act at the level of the spinal cord to reduce spinal proinflammatory cytokines. This possibility is supported by general anti-inflammatory actions observed when given systemically.

For the first time, the current report demonstrates that DPX can suppress overexpression of IFN-γ in the spinal cord following CCI. Sonekatsu, et al. ^65^ found that increases in IFN-γ in the spinal cord can activate microglia leading to increased NMDA-induced excitatory post-synaptic potentials in dorsal horn neurons, which is believed to contribute to chronic neuropathic pain ^66,67^. The attenuation of allodynia in CCI mice accompanies the alteration of dorsal horn spinal proinflammatory cytokines, TNFα, IL-1β, and IFN-γ in the central nervous system while leaving peripheral cytokines intact.

Unexpectedly, the hypothalamus, and not the anterior cingulate cortex nor the amygdala, revealed significant TNFα and IL-1β changes compared to other cytokines in response to CCI and DPX. However, in line with prior work, CCI-induced allodynia creates a stressor, and stress increases IL-1β and TNFα along with microglial and astrocyte activation in the hippocampus ^68^. Interestingly, a local i.t. application blunted IL-1β and TNFα protein expression, supporting the possibility that pain alleviation may reduce stress (i.e. stress from allodynia) via engaging spinal-to-brainstem and limbic circuitry. Despite the impact of CCl-induced allodynia on hypothalamic cytokines, the sympathetic influence of the hypothalamus appeared to remain unaltered, as no reliable changes in splenic cytokine mRNA or norepinephrine expression was observed.

Dexpramipexole offers a new therapeutic target and non-opioid approach to treat chronic pain in light of evidence that current non-opioid analgesics often provide suboptimal pain relief due to mainly targeting neuronal sensory pathways. These drugs do not effectively address the neuroimmune and inflammatory mechanisms that lead to sustained chronic pain ^69,70^. It is important to reiterate that many preclinical studies examining persistent chronic pain states show that such neuropathies are driven by the activation of microglia and astrocytes in the spinal cord and brain. Given that many analgesics mainly target neuronal action while leaving the neuroimmune and inflammatory response intact, they may not fully address the complex mechanisms that underlie neuropathic and chronic pain ^69,70^. Thus, DPX may address this treatment gap through its action on nociceptors as well as affecting pronociceptive cytokine expression in key central nervous system structures. In addition, DPX may act as a potential adjunctive therapy when co-administered with opioid compounds, as a growing body of evidence demonstrates that repeated opioid administration may increase pronociceptive cytokine production through opioid activation of the immune cell receptor, toll-like receptor 4, which is expressed on spinal astrocytes and microglia. Several reports have demonstrated that opioid-induced glial activation may play a significant role in the development of opioid tolerance. While speculative, DPX as a therapeutic adjunct to opioid therapy may be ideal due to its role in diminishing opioid tolerance with prolonged use ^71^.

Our data demonstrates that after administration of intravenous DPX, a robust reversal of allodynia to baseline levels occurs. Independent from the action of DPX on Nav_1.8_, DPX may cross the blood brain barrier via lipid-mediated free diffusion to act in the spinal cord resulting in a reduction of glially-mediated IFN-γ release, while leaving neurons intact ^72^. Studies show that spinal microglia are commonly activated following peripheral nerve injury, and in turn increase expression of neurotransmitter receptors and proinflammatory cytokines thereby contributing to enhanced pain processing ^72^, and activation of spinal microglial IFN-γ receptor is strongly linked to the development of neuropathic pain. Thus, systemic administration of DPX may act not only at the Nav_1.8_ on peripheral nociceptors, but also at the spinal level to reduce pro-nociceptive cytokines resulting in pain alleviation from a peripheral nerve injury.

## Conclusions

The current data replicates prior reports demonstrating that systemic application of DPX reverses allodynia from sciatic nerve injury in female mice. At the level of the spinal cord, intrathecal application DPX reduces protein expression of the pronociceptive cytokine, IFN-γ, as well as modest reductions in spinal protein and dorsal root ganglia mRNA for IL-1β and TNFα in mice that also demonstrated a full reversal of tactile allodynia following nerve injury. The action of DPX as an anti-proinflammatory agent in the central nervous system while also acting to block nociceptive Nav_1.8_ actions offers a safe, unique and layered approach for treating chronic pain. Importantly, opioids have been long used to manage chronic pain ^73^ but pose a risk of adverse side effects including, but not limited to, gastrointestinal problems, immunosuppression, opioid tolerance, substance abuse disorder, opioid-induced hyperalgesia, and overdose. ^74,75^ Compounds with high safety profiles like DPX are worthy of further investigation, as it is devoid of opioid actions can help mitigate risks and offer pain relief in a clinical setting.

## Figures and Tables

**Table 1:** primer identification for all targets quantified in spleen and DRG.

Target Name	TaqMan Primer Assay ID (ThermoFisher Scientific)

**IL-1β**	Mm00434228_ml
**TNF-α**	Mm00443258_ml
**IL-10**	Mm01288386_ml

**Table 2:** Protein expression levels in picogram (pg) target protein/microgram (ug) total protein in specific limbic brain regions.

	Group				Region				
		ACC		DSC		HYP		AMG	
		AVE ($\bar{\text{x}}$)	SD (σ)	$\bar{\text{x}}$	σ	$\bar{\text{x}}$	σ	$\bar{\text{x}}$	σ
**IL-6**	SHAM + VEH	3.706E-03	2.100E-03	4.506E-03	4.046E-03	7.079E-03	2.234E-03	3.680E-03	2.675E-03
	SHAM + DPX	5.235E-03*	2.920E-03	3.045E-03	2.213E-03	4.102E-03	9.328E-04	3.914E-03	1.962E-03
	CCI + VEH	3.274E-03	1.492E-03	2.450E-03	8.188E-04	3.078E-03	1.655E-03	3.591E-03	1.818E-03
	CCI+DPX	5.046E-03*	1.586E-03	5.932E-03	3.981E-03	6.986E-03	2.547E-03	4.260E-03	2.136E-03
**INF-γ**	SHAM + VEH	1.520E-05	6.314E-06					2.005E-05	6.524E-06
	SHAM + DPX	1.994E-05	4.077E-06					2.064E-05	6.623E-06
	CCI + VEH	1.671E-05	5.350E-06					2.030E-05	7.825E-06
	CCI+DPX	2.036E-05	3.763E-06					2.217E-05	4.918E-06
**IL-1β**	SHAM + VEH	7.548E-05	1.831E-05					2.689E-05	1.551E-05
	SHAM + DPX	7.199E-05	4.378E-05					2.765E-05	1.656E-05
	CCI + VEH	1.177E-04	6.152E-05					2.700E-05	7.747E-06
	CCI+DPX	1.012E-04	3.148E-05					4.639E-05	3.032E-05
**IL-10**	SHAM + VEH	1.865E-04	4.336E-05	3.361E-04	4.037E-04	2.838E-04	2.646E-04		
	SHAM + DPX	3.857E-04^^^	1.656E-04	1.990E-04	2.067E-05	5.086E-04	2.224E-04		
	CCI + VEH	2.370E-04	1.117E-04	3.226E-04	2.001E-04	4.368E-04	2.373E-04		
	CCI+DPX	2.298E-04	9.183E-06	1.912E-04	1.025E-04	4.910E-04	2.111E-04		
**TNF-α**	SHAM + VEH	6.872E-05	2.541E-05			1.163E-04	4.889E-05	
	SHAM + DPX	8.940E-05	2.132E-05			6.422E-05*	2.667E-05		
	CCI + VEH	7.368E-05	2.327E-05			1.517E-04	6.773E-05		
	CCI+DPX	6.942E-05	5.139E-06			7.836E-05*	7.808E-06		

Select targets analyzed: interleukin-6 (IL-6), interferon gamma (INF-γ), interleukin-1 beta (IL-1β) interleukin-10 (IL-10), and tumor necrosis factor alpha (TNF-α). All results reflect 2-way ANOVA main effects of surgery and drug, and interactions of surgery and drug.

* and ^^^ denote p≤0.05.

**Table 3.** mRNA expression levels (fold change) for cytokines in spleen and norepinephrine (NE) protein expression levels in pg target protein/ug total protein in the adrenal glands.

Target	Group	Region	
		Spleen mRNA (fold change)	
		$\bar{\text{x}}$	σ
IL-10	SHAM + VEH	1.027E+00	4.636E−01
	SHAM + DPX	9.241E−01	8.359E−01
	CCI + VEH	6.016E−01	3.012E−01
	CCI+ DPX	8.535E−01	8.106E−01
			
IL-1β	SHAM + VEH	1.079E+00	7.746E−01
	SHAM + DPX	6.936E−01	3.519E−01
	CCI + VEH	8.743E−01	6.723E−01
	CCI+ DPX	8.116E−01	8.824E−01
			
TNF-α	SHAM + VEH	1.146E+00	1.208E+00
	SHAM + DPX	6.525E−01	3.658E−01
	CCI + VEH	5.872E−01	2.434E−01
	CCI+ DPX	8.102E−01	3.862E−01
			
		ADR (pg NE/mg total protein)	
NE	SHAM + VEH	7.112E+00	5.912E+00
	SHAM + DPX	6.179E+00	1.768E+00
	CCI + VEH	4.495E+00	2.732E+00
	CCI+ DPX	6.697E+00	4.777E+00

All results reflect 2-way ANOVA. No differences were found between groups in either peripheral region.
